# Alteration of Forkhead Box O (Foxo4) Acetylation Mediates Apoptosis of Podocytes in Diabetes Mellitus

**DOI:** 10.1371/journal.pone.0023566

**Published:** 2011-08-17

**Authors:** Peter Y. Chuang, Yan Dai, Ruijie Liu, Helen He, Matthias Kretzler, Belinda Jim, Clemens D. Cohen, John C. He

**Affiliations:** 1 Division of Nephrology, Department of Medicine, Mount Sinai School of Medicine, New York, New York, United States of America; 2 Department of Nephrology, Shanghai Jiaotong University Affiliated First People's Hospital, Shanghai, China; 3 James J. Peters VA Medical Center, Bronx, New York, United States of America; 4 Division of Nephrology, Department of Internal Medicine, University of Michigan, Ann Arbor, Michigan, United States of America; 5 Division of Nephrology, Department of Medicine, Jacobi Medical Center, Albert Einstein College of Medicine, Bronx, New York, United States of America; 6 Division of Nephrology and Institute of Physiology, University of Zurich, Zurich, Switzerland; University of Houston, United States of America

## Abstract

The number of kidney podocytes is reduced in diabetic nephropathy. Advanced glycation end products (AGEs) accumulate in patients with diabetes and promote the apoptosis of podocyte by activating the forkhead box O4 (Foxo4) transcription factor to increase the expression of a pro-apoptosis gene, *Bcl2l11*. Using chromatin immunoprecipitation we demonstrate that AGE-modified bovine serum albumin (AGE-BSA) enhances Foxo4 binding to a forkhead binding element in the promoter of *Bcl2lll*. AGE-BSA also increases the acetylation of Foxo4. Lysine acetylation of Foxo4 is required for Foxo4 binding and transcription of *Bcl2l11* in podocytes treated with AGE-BSA. The expression of a protein deacetylase that targets Foxo4 for deacetylation, sirtuin (Sirt1), is down regulated in cultured podocytes by AGE-BSA treatment and in glomeruli of diabetic patients. SIRT1 over expression in cultured murine podocytes prevents AGE-induced apoptosis. Glomeruli isolated from diabetic *db/db* mice have increased acetylation of Foxo4, suppressed expression of *Sirt1*, and increased expression of *Bcl2l11* compared to non-diabetic littermates. Together, our data provide evidence that alteration of Foxo4 acetylation and down regulation of *Sirt1* expression in diabetes promote podocyte apoptosis. Strategies to preserve *Sirt1* expression or reduce Foxo4 acetylation could be used to prevent podocyte loss in diabetes.

## Introduction

Diabetic nephropathy (DN) is the leading cause of renal failure in the western world. It is thought that hyperglycemia activates multiple downstream signaling pathways in the diabetic kidney leading to extracellular matrix accumulation, endothelium dysfunction, glomerular hyperfiltration, oxidant stress, and advanced glycation end products (AGEs) formation, which all contribute to the development of DN [1]. Reduction in the density of kidney podocytes occurs early in the development of DN and correlates with the progression of DN [2] [3] [4] [5] [6]. Apoptosis of podocytes is a potential cause of reduction in podocyte number in diabetes. Oxidant stress from hyperglycemia, angiotensin II, and AGE are known to induce podocyte apoptosis in diabetes [7], [8], [9].

AGE accumulates in diabetes and reduction of AGE formation ameliorates the development of DN [10]. We have found previously that a member of the forkhead box class O (Foxo) family of transcription factors, Foxo4, is required for AGE-induced podocyte apoptosis [9]. The Foxo family of transcription factors (TFs) is involved in the regulation of oxidant stress resistance, apoptosis, cell cycle inhibition, cellular metabolism, and DNA damage repair (reviewed in [11]). The activity of Foxo is regulated by post-translational modifications, including phosphorylation, ubiquitylation, and acetylation. Change in the acetylation status of a member of the Foxo family, Foxo3, has been shown to alter the differential expression of Foxo target genes in a context-specific manner [12]. In this study, we examined the role of Foxo4 acetylation in podocyte apoptosis in vitro and in vivo. We find that AGE increases Foxo4 acetylation and suppresses the expression of the Sirt1 protein deacetylase in kidney podocytes. Acetylated Foxo4 promotes the expression of a pro-apoptosis gene *Bcl2l11* (also known as Bim) and leads podocyte apoptosis.

## Methods

### Cells and Antibodies

A murine podocyte cell line from Dr. Peter Mundel (Massachusetts General Hospital, Boston, Massachusetts) was cultured as previously described [9]. The following antibodies were used: a mouse monoclonal and a rabbit polyclonal Sirt1 antibody (Millipore, Billerica, MA), a goat polyclonal Foxo4 antibody (Santa Cruz Biotechnology Inc., Santa Cruz, CA), a rabbit polyclonal Foxo4 antibody (Cell Signaling Laboratory, Danvers, MA), a rabbit polyclonal acetyl-lysine antibody (Cell Signaling Laboratory), a rat monoclonal Bim antibody (Millipore), and a mouse monoclonal synaptopodin antibody (Fitzgerald Industries, Acton, MA). Endotoxin-free AGE-BSA was prepared from endotoxin-free, lyophilized BSA (fraction IV; Sigma, St Louis, MO) and D-glucose, as described previously [13], [9].

### Experimental protocol for diabetes mellitus

Animal studies were performed in accordance with the guidelines of and approved by the Institutional Animal Care and Use Committee at the Mount Sinai School of Medicine (Protocol 08-0106-00001-01). Researchers handling animals were trained and certified according to the guidelines of the American Association for Laboratory Animals. Diabetic *db/db* mice on the C57BLKS background were purchased from Jackson Laboratory, Bar Harbor, ME and raised in the animal facilities at the Mount Sinai School of Medicine (New York, NY) according to standardized specific pathogen-free conditions. Mice were maintained on standard mouse chow and allowed free access to tap water. Four hour fasting serum glucose level was checked every week from 6 weeks of age. Animals with random serum glucose levels consistently above 250 mg/dl after 6 weeks of age were included in the study.

### Podocytes with stable over expression of Sirt1

A human Sirt1 cDNA clone purchased from Thermo Scientific (Huntsville, AL, USA) was PCR amplified and inserted between *BamHI* and *XhoI* of the pDNR-1r vector (Clontech). The Sirt1-pDNR-1r construct was digested with *BamHI* and *EcoRV* then inserted into a *gag-*, *pol-*, and *env-* deficient lentivector construct, *VVE/BBW* (a gift of Dr. G Luca Gusella, Mount Sinai School of Medicine) to generate the *VVE/BBW-Sirt1* lentivector. Lentiviral particles used to transduce Sirt1 in podocytes were produced in 293T/17 cells (from ATCC; number CRL-11268) by co-transfection of *VVE/BBW-Sirt1* with a packaging and an envelope plasmid. Podocytes with stable Sirt1 expression (Sirt1) were selected using blasticidin (8 ug/ml). Podocytes infected with the backbone lentivector (BB) served as infection controls.

### Podocyte with stable over expression of a Foxo4 acetylation mutant

A FLAG-Foxo4 construct (Addgene, Cambridge, MA) was used as the template to generate a Foxo4 acetylation mutant by PCR-mediated mutagenesis using QuikChange® Site-Directed Mutagenesis Kit (Strategene, La Jolla, CA). Three lysine residues at 186, 189 and 407 were mutated to alanine (Foxo4 KA Mut). The mutations in Foxo4 KA Mut was confirmed by DNA sequencing. The mutant was inserted into the VVE/BBW lentivector and lentiviral particles were generated to tranduce podocytes. Podocytes with stable over expression of Foxo4 KA Mut were selected using blasticidin.

### Podocytes with shRNA-mediated knockdown of Sirt1

Five pKLO.1 replication-incompetent lentiviral vectors containing specific a shRNA targeting Sirt1 [14] were purchased from Open Biosystems/Thermo Fisher Scientific (Huntsville, AL) and tested for Sirt1 knockdown in cultured podocytes. Lentivral vector were co-transfected with a packaging and an envelope vector into 293T cells to generate viral particles. Viral particles were used to infect conditionally immortalized podocytes cultured at 33°C. Transduced podocytes were selected using puromycin and cultured at 37°C for at least 7 days prior to experiments. Of the four clones of shRNA targeting Sirt1, clone TRCN0000039294 had the most significant knockdown of Sirt1 (>70% knockdown compared to a scrambled shRNA), which was used in subsequent experiments.

### Western Blotting

Cell lysates were subjected to 8–12% SDS-PAGE prior to transfer to nitrocellulose membranes as previously described [9]. Densitometric measurements of the band intensities on scanned images were made using ImageJ [15].

### Archival human kidney biopsy samples

Archival human kidney biopsies were collected at Jacobi Medical Center, Bronx, New York as part of an exempted protocol (Differential Protein Expression in Nephrotic Diseases), which was approved by the Einstein Institutional Review Board of the Albert Einstein College of Medicine of Yeshiva University. Since all clinical data were de-identified, no consent was required. Samples included 4 with DN (3 mild DN and 1 nodular DN), 2 with minimal change disease (MCD), and 2 with no apparent kidney disease on histology (Normal).

### Immunostaining of paraffinized kidney sections

Kidney sections from mice were prepared as described [16]. Sirt1 immunostaining was performed using a rabbit polyclonal Sirt1 antibody. A citrate-based antigen retrieval solution was used. Endogenous peroxidase was blocked in H_2_O_2_. Non-specific protein binding was blocked with 3% BSA. Sections were incubated with the Sirt1 antibody (diluted 1∶30 in 3% BSA) at 4°C for overnight. After washing, sections were incubated with an anti-rabbit biotinylated secondary antibody at room temperature, and then with the avidin–biotin–peroxidase complex (Vectastin Elite ABC Kit, Vector Laboratories). The reaction products were developed using the 3, 3′-diaminobenzidine substrate from Vector Laboratory, mounted with a glass coverslip, and photographed using a Zeiss Axioplan2 microscope with a Q-imaging MP3.3 RTV camera. The number of podocytes with positive Sirt1 staining was counted in 10 glomeruli from each biopsy case. Podocytes in histologic sections were identified based on morphology and their location relative to the glomerular basement and Bowman's space as previously described [16].

### Glomeruli isolation by iron oxide perfusion

Glomeruli were isolated by Fe_3_O_4_ perfusion as previously described [17]. Mice were perfused with Hank's buffered salt solution (HBSS) containing 2.5 mg/ml Fe_3_O_4_ (Sigma Aldrich, St Lois MO) and 1% BSA. Kidneys were digested with collagenase A and DNase I in HBSS at 37°C for 30 min, passed through 100 µm cell strainers, rinsed with HBSS, and collected using a magnet (Dynal MPC, Invitrogen, Norway). The purity of the glomeruli isolate was confirmed by light microscopy and by Western blot for synaptopodin expression.

### Quantification of podocyte apoptosis by flow cytometry

Podocyte apoptosis was quantified by flow cytometry after annexin V-fluorescein isothiocyanate (FITC) labeling following manufacturer's protocol (Annexin V-FITC Apoptosis Detection Kit I, BD Bioscience, San Jose, CA, USA).

### Chromatin immunoprecipiation (ChIP)

ChIP was performed with modifications of an existing protocol [18]. For each ChIP reaction, 5×10^7^ podocytes were used. Cells were serum starved for 16 h prior to treatment with either 100 µg/ml of AGE-BSA or BSA as control and 30 mM of glucose (HG) or 30 mM of mannitol (Man) as osmotic control. After 2 h of treatment, cells were crosslinked for 10 min, then quenched with a glycine buffer. Chromatin was extracted, then sonicated using a Misonix 3000 Sonicator with a microtip (16 cycles at power 6 with 20 s of sonication and 60 s of rest). Immunoprecipitation was performed using a goat anti-Foxo4 antibody linked to tosylactivated magnetic beads (Dynabeads M-450 Tosylactivated, Invitrogen). Normal goat IgG tosyl-linked to Dynabeads was used as a control. Cross-linking was reversed by incubation at 65°C for 8 h followed by RNAse A and proteinase K digestions. DNA from immunoprecipitated chromatin was purified using the Qiagen PCR purification kit (Qiagen, Hilden, Germany). Forkhead binding elements (FBE) in the promoter of *Bcl2l11* were predicted using a commercial software tool (MatInspector, Genomatix, Ann Arbor, MI). A pair of oligonucleotide primers flanking a predicted FBE in the promoter of *Bcl2l11* was synthesized. (*FBE_for_*: CTC TAT TGT GAC GCA CTT ACT A and *FBE_rev_*: CTT TCC TTC AGG ACA AAC TTG A). Primer pairs targeting −2241 to −2043 of the translational start site were used to detect nonspecific immunoprecipitation (*NS_for_*: GGA GTC GGG TCG AGG TCG CT; *NS_rev_*: GGA CGC ACT ACC GGC ACC AC). Amount of immunoprecipitated DNA was compared by end point PCR with the following thermoprofile: 94°C×3 min, 40 cycles of 94°C×15 s, 54°C×45 s and 72°C×45 s, and followed by 72°C×5 min. Quantitative realtime PCR using ChIP DNA samples as templates and *FBE_for_* and *FBE_rev_* primer pairs were performed. Cp values from IgG and Foxo4 antibodies immunoprecipitated templates were normalized to Cp values from the corresponding Input DNA templates. Percentage of Input DNA template was calculated using the formula: percentage of Input = 100×2^(Cp of Input−Cp of IgG or Foxo4)^.

### Quantitative real-time PCR

Total RNA was extracted from isolated glomeruli or cultured immortalized murine podocytes using either RNeasy Mini Kit (Qiagen) or TRIzol reagent (Invitrogen), respectively. cDNA were reverse transcribed from total RNA using SuperScript III First-Strand Synthesis SuperMix (Invitrogen). PCR was performed using Sybr Green Master Mix (Applied Biosystems, Foster City, CA) and the Applied Biosystems 7900HT Fast Real-Time PCR System. The 2^−ΔΔCp^ method was used for the calculation of relative gene expression. Primers for realtime PCR were designed using Primer-Blast (NCBI, Bethesda, MD) to span at least one intron. Gene expression was normalized to GAPDH as a housekeeping gene and presented as relative expression compared to a calibrator sample.

### Detection of endogenous Foxo4 acetylation by Western Blotting

Total protein was extracted from cells or tissues using a lysis buffer containing inhibitors of Class I, II and III deacetylases (50 mM Tris HCl, pH 7.4, 150 mM NaCl, 1 mM EDTA, 1% Triton X-100, 10 uM trichostatin, 5 mM nicotinamide). Foxo4 was immunoprecipitated using a goat polyclonal anti-Foxo4 antibody. Acetylation of Foxo4 was assessed by Western blotting using a rabbit anti-acetyl-lysine antibody. Successful immunoprecipitation of Foxo4 was confirmed by blotting with a rabbit polyclonal Foxo4 antibody.

### Fluorescence-based Foxo4 acetylation assays

HEK-293 (from ATCC) transfected with the FLAG-Foxo4 construct were serum starved for 18 hrs prior to treatment. Cells were lysed and FLAG-Foxo4 in the protein lysate was pulled down using anti-FLAG M2 beads (Sigma, St Louis, MO). Acetylation of FLAG-Foxo4 was measured using a commercially available kit (HAT Inhibitor Screening Assay Kit, Cayman Chemical Company, Ann Arbor, MI) with some modifications to the protocol: To determine the relative amount of acetylated lysine residues, immunoprecipiated Flag-Foxo4 were incubated with acetyl CoA and an acetylase (P300/CBP-associated factor) for 5 min at room temperature. The amount of free CoA was determined using 7-diethylamino-3-(4′-maleimidylphenyl)-4-methyl-coumarin (CPM), which conjugates with free CoA. The amount of CoA-modified CPM was measured using a plate reader (excitation wavelength of 320 nM and emission wavelength of 450 nm). The relative fluorescent unit (RFU) measured is indirectly proportional to the acetylation of FLAG-Foxo4. Relative Foxo4 acetylation at a given time point is calculated using the following formula: RFU_time 0_/RFU_time **x**_.

### Activated Caspase 3 assay

A commercially available ELISA (R&D Systems, Minneapolis MN) for active caspase 3 (Quantikine) was used to assess apoptosis. Podocytes (1×10^5^ cells/condition) transduced and selected for stable expression of shRNAs targeting either Sirt1 or scrambled sequence were cultured on type I collagen-coated tissue culture plates for 7 days prior treatment with either 30 mM of mannitol or D-glucose for 16 h in serum free medium. For each experimental condition, active caspase 3 level was measured in duplicates. Results were normalized to cells transduced with the scrambled shRNA and treated with mannitol.

### Sirt1 expression analysis by cDNA arrays

Data were generated in the context of the European Renal cDNA Bank–Kröner-Fresenius Biopsy bank (ERCB-KFB; see Acknowledgment for participating centers). Human renal biopsy specimens were obtained after written consent and approval of the ethics committee and in the frame of the ERCB approved by the specialized subcommittee for internal medicine of the Cantonal Ethics Committee of Zurich (Kantonale Ethikkommission Zürich). The methods of microdissection and microarray experiments have been published for glomeruli and tubulointerstitium previously [19], [20]. The array data were analyzed using ChipInspector (Genomatix, Munich, Germany), a probe sequence-specific analysis software for microarray data [20]. All microarray data are MIAME compliant as detailed on the MGED Society website http://www.mged.org/Workgroups/MIAME/miame.html. Microarray data will be deposited in Gene Expression Omnibus. A main part of the microarray data is available online at http://www.nephromine.org.

### Statistical analysis

Values are expressed as means ± S.E.M. Differences between means were calculated by t-test. In all analyses, the null hypothesis was rejected at 0.05. Statistical analyses were performed using either Microsoft Excel or Prism Stat program (GraphPad Software, Inc., San Diego, CA). For comparison of measurements from more than 2 groups of samples ANOVA was used.

## Results

### AGE-BSA induced podocyte apoptosis by promoting Foxo4-mediated expression of *Bcl2l11*

**Figure 1:**
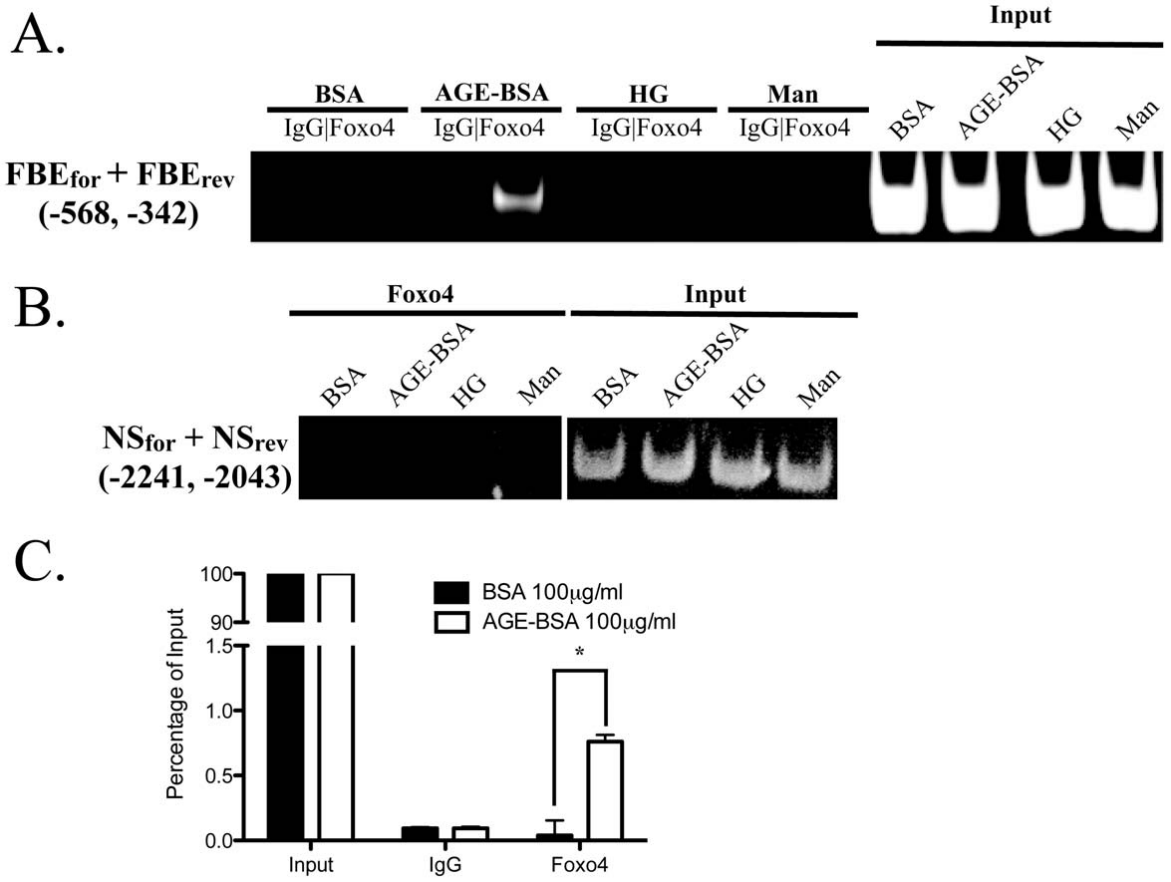
Binding of Foxo4 to the *Bcl2l11* promoter. Conditionally immortalized podocytes were treated with either AGE-BSA (100 µg/ml) or BSA (100 µg/ml) as control and 30 mM of hyperglycemia (HG) or 30 mM of mannitol (Man) as osmotic control for 2 h. Binding of Foxo4 to a predicted forkhead binding elementer (FBE) in the promoter region of *Bcl2l11* was characterized by chromatin immunoprecipiation (ChIP) using either an anti-Foxo4 antibody (Foxo4) or normal goal IgG (IgG). (A) Binding was assessed using primers flanking FBE (FBE_for_ and FBE_rev_) and immunoprecipitated DNA samples as PCR templates. (B) Primers flanking a region −2241 to −2043 upstream of the translational start site were used in a PCR reaction with Foxo4 immunoprecipitated DNA as templates to assess non-specific immunoprecipitation. Representative images of products of endpoint PCR resolved on agarose gels are shown. (C) Data of quantitative realtime PCR of ChIP DNA using primers flanking the FBE. Results from 3 independent sets of ChIPs. * *p*<0.05.

We previously found that AGE-BSA increased the expression of *Bcl2l11*, which is a Foxo-target gene involved in apoptosis response [9]. To confirm that Foxo4 binds to the promoter of Bcl2l11 in AGE-BSA treated podocytes, we examined the binding of FOXO4 to a predicted Foxo binding element (FBE) upstream of *Bcl2l11* by ChIP. AGE-BSA treatment of cultured podocytes enhanced Foxo4 binding to the FBE of *Bcl2l1l* without any nonspecific binding to a region (−2241 to −2043) upstream of the *Bcl2l11* transcriptional start site that does not contain FBE (Fig. 1A, 1B). Quantitative real time PCR analysis of immunoprecipitated DNA demonstrated that AGE-BSA significantly increased the binding of Foxo4 to the FBE of *Bcl2l11* compared to BSA (0.76±0.09% vs. 0.04±0.2%, p<0.01, Fig. 1C). Nonspecific binding using normal goat IgG was minimal (Fig. 1A).

### AGE-BSA increases Foxo4 acetylation and Bcl2l11 expression

**Figure 2:**
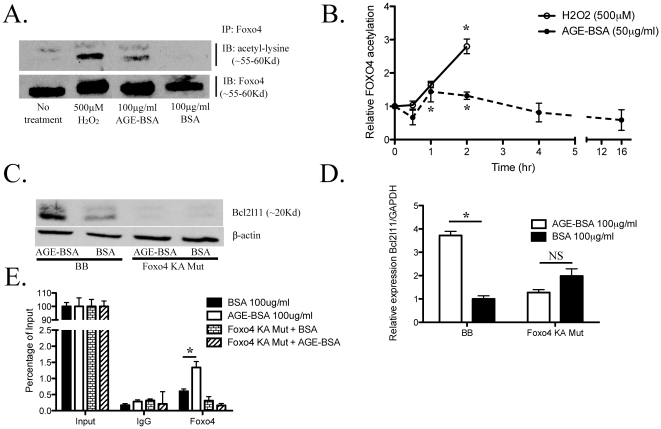
Acetylation of Foxo4 is required for AGE-BSA-induced Bcl2l11 acetylation. (A) Acetylation of endogenous Foxo4 in cultured podocytes treated with 500 µM of H_2_O_2_, 100 µg/ml of AGE-BSA or 100 µg/ml of BSA for 2 h was assessed by gel electrophoresis of immunoprecipitating Foxo4, followed by immunoblotted with an anti-acetyl-lysine antibody to detect acetylated Foxo4. Immunoblotting with an anti-Foxo4 antibody confirms the immunoprecipitation of Foxo4. (B) H_2_O_2_- and AGE-BSA-induced acetylation of a FLAG-Foxo4 fusion protein was confirmed using a fluorescence-based *in vitro* acetylation assay. 293 cells transfected with a FLAG-Foxo4 fusion construct and serum starved for 16–18 hrs were treated with either H_2_O_2_ (500 µM) or AGE-BSA (50 µg/ml) at 0, 0.5, 1, 2, 4, or 16 hr. (Relative Foxo4 acetylation with AGE-BSA at 1 h is 1.36±0.12 and at 2 h is 1.32±0.11, *p<0.05, n = 4; with 500 µM of H_2_O_2_ at 2 hr is 2.80±0.22 *p<0.05, n = 3). The expression of Bcl2l11 in podocytes with stable over expression of the Foxo4 KA mutant (Foxo4 KA Mut) was compared to control podocytes (BB) after AGE-BSA (100 µg/ml) treatment for either 24 h (in RT PCR experiments) or 72 h (for Western Blotting experiments). Foxo4 KA Mut over expression abrogated AGE-BSA induced Bcl2l11 expression as characterized by Western blotting (C) and RT PCR (D). Binding of Foxo4 to the FBE in the *Bcl2l11* promoter was reduced in podocytes with Foxo4 KA Mut over expression as characterized by ChIP of Foxo4 followed by RTPCR using primers flanking FBE (FBE_for_ and FBE_rev_).

Since oxidant stress is known to increase Foxo3 and Foxo4 acetylation [12], [21], [22] and AGE promotes intracellular oxidant stress [23], we tested the effects of AGE-BSA (100 µg/ml) on the acetylation of Foxo4 in murine podocytes. High dose H_2_O_2_ (500 µM) was used as a positive control for Foxo4 acetylation. AGE-BSA treatment increased Foxo4 acetylation, but to a lesser extent than 500 µM H_2_O_2_ (Fig. 2A). Acetylation of FLAG-Foxo4 in response to AGE-BSA and H_2_O_2_ treatments was confirmed in 293 cells using a fluorescence-based acetylation assay (Fig. 2B). Maximal acetylation occurred between 1 to 2 h after AGE-BSA stimulation (fold increase in acetylation at 1 h and 2 h are 1.36±0.12 and 1.32±0.11, p<0.05). H_2_O_2_ (500 µM) is a more potent stimulator of FLAG-Foxo4 acetylation than AGE-BSA at 2 h of treatment (2.80±0.22 fold increase in acetylation, p<0.05). The effect of hyperglycemia on Foxo4 acetylation was also tested. No significant change in Foxo4 acetylation was observed between 30 mM of D-glucose and 30 mM of mannitol. (Data is not shown).

### Acetylation of Foxo4 is required for AGE-mediated induction of Bcl211 expression

To determine the role of Foxo4 acetylation on Bcl2l11 expression we over expressed a Foxo4 mutant (Foxo4 KA Mut), which is mutated at three key lysine residues that are known to be acetylated, and examined the effect of Foxo4 KA Mut on Bcl2l11 expression in response to AGE-BSA treatment. Foxo4 KA Mut prevented AGE-BSA-induced Bcl2l11 expression as determined by western blotting and RT PCR (Figure 2C and 2D). Furthermore, Foxo4 KA Mut binding to the Bcl2l11 promoter in response to AGE-BSA treatment was reduced in podocytes confirming that acetylation of Foxo4 is essential for its binding and transactivation of Bcl2l11 (Figure 2E).

**Figure 3:**
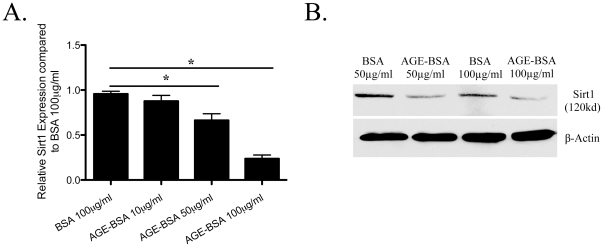
Levels of *Sirt1* mRNA and protein. A: Total mRNA extracted from podocytes treated with AGE-BSA (10 µg/ml, 50 µg/ml, and 100 µg/ml) or BSA (100 µg/ml) for 4 h was used to quantify the *Sirt1* mRNA level by realtime PCR. Sirt1 expression was significantly reduced with 50 µg/ml and 100 µg/ml of AGE-BSA compared to 100 µg/ml of BSA. (n = 3, * p<0.05) B: Sirt1 is reduced by AGE-BSA (50 and 100 µg/ml) compared to BSA (50 and 100 µg/ml) treatment as assessed by Western blotting. A representative blot is shown here.

*Sirt1 expression is suppressed by AGE-BSA*—Sirt1 is a protein deacetylase that binds and de-acetylates Foxo4 [22]. Since Foxo4 acetylation is enhanced by AGE-BSA, we tested the hypothesis that AGE-BSA suppresses Sirt1 to promote the accumulation of acetylated Foxo4. Sirt1 mRNA and protein levels were quantified by RT PCR and Western blotting, respectively, in podocytes treated with AGE-BSA. AGE-BSA suppressed Sirt1 mRNA expression significantly at 50 and 100 µg/ml (Fig. 3A). Down regulation of Sirt1 by AGE-BSA at 50 and 100 µg/ml was further confirmed by Western blotting (Fig. 3B).

### Sirt1 expression is reduced in glomeruli of patients with diabetic nephropathy

**Figure 4:**
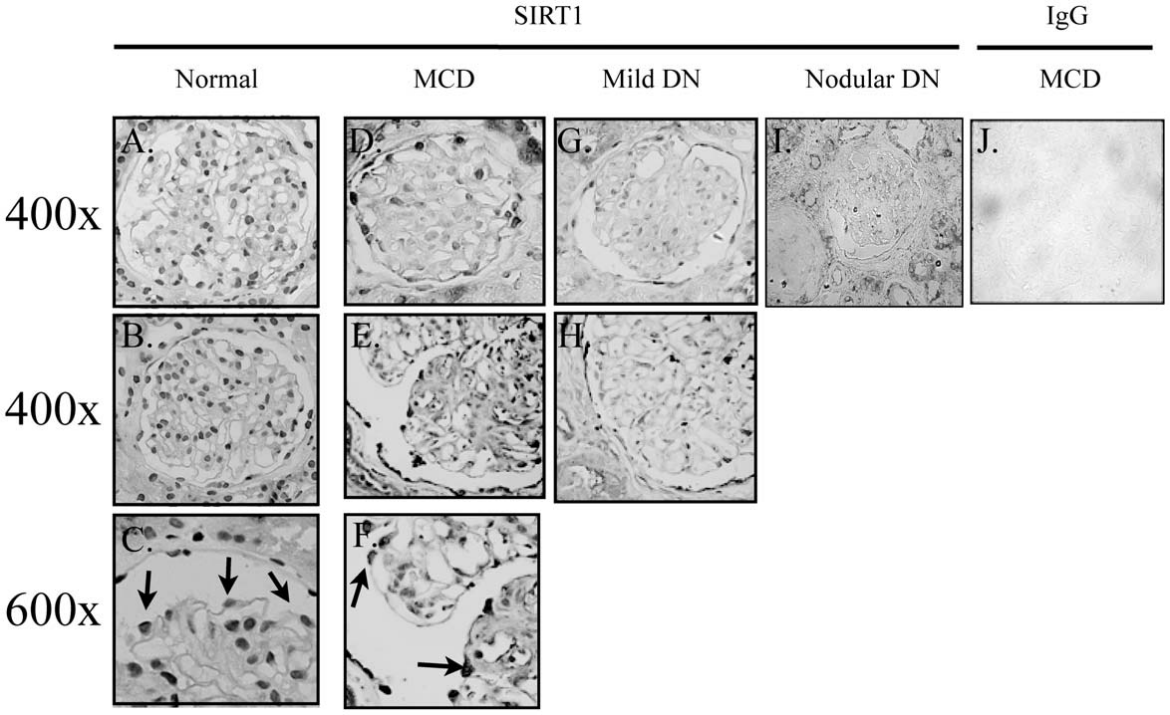
Sirt1 immunostaining. Archival renal biopsy samples from patients with no apparent renal disease (normal), minimal change disease (MCD), mild diabetic nephropathy (mild DN) and nodular DN were used for Sirt1 immunostaining. Representative staining are shown for Normal (A, B, C) minimal change disease (D, E, and F), mild DN (G and H) and nodular DN (I). Normal rabbit IgG labeling of MCD (J) displayed minimal nonspecific staining. A higher magnification view (600×) of cells near the periphery of a glomerulus shows the nuclear pattern of Sirt1 staining (arrows in C and F).

Since the level of AGE is elevated in the serum of patient with diabetes and Sirt1 expression in cultured podocytes is suppressed by AGE, we examined the expression of Sirt1 in the kidneys of patients with diabetes. Sirt1 immunostaining of archival kidney biopsy sections from patients with Normal, MCD, DN (mild DN and nodular DN) revealed that Sirt1 expression localizes to the nuclei of both tubular and glomerular cells (Fig. 4). In the glomeruli, Sirt1 antibody labeled cells near the periphery of the glomeruli, which are thought to be podocytes (Fig. 4C and 4F, arrows). The intensity of Sirt1 staining in glomerular cells is reduced in kidney sections from patients with mild DN compared to those from MCD and Normal and further reduced in sections from patients with nodular DN (Fig. 4D to 4I). Sirt1 staining in the tubules did not appear to be different in patients with MCD and DN. The estimated average of Sirt1-positive podocytes per glomeruli in Normal 9.0±1.1, in MCD is 8.0±0.25, in DN (mild+nodular) DN is 2.7±0.37. A more quantitative assessment of the expression of Sirt1 in patients with DN was made by querying the ERCB Consortium for Sirt1 mRNA expression in microdissected glomeruli from an European cohort of diabetic patients [24]. We compared mRNA levels of *Sirt1* (EnTrez Gene 23411; Representative public ID: NM_012238) in patients with DN compared to those from living donors of kidney transplantation (LD) in two independent microarray experiments (DN: n = 7 each; LD: n = 4 and 18, respectively). The expression of Sirt1 in patients with DN from two different sets of experiments was significantly reduced by 36% and 27%, respectively. There was no statistical difference in the expression of Sirt1 in the tubulointerstitial compartment, which is consistent with our observation from Sirt1 immunostaining of kidney biopsy sections.

### Sirt1 over expression protects against AGE-BSA-induced podocyte apoptosis

**Figure 5:**
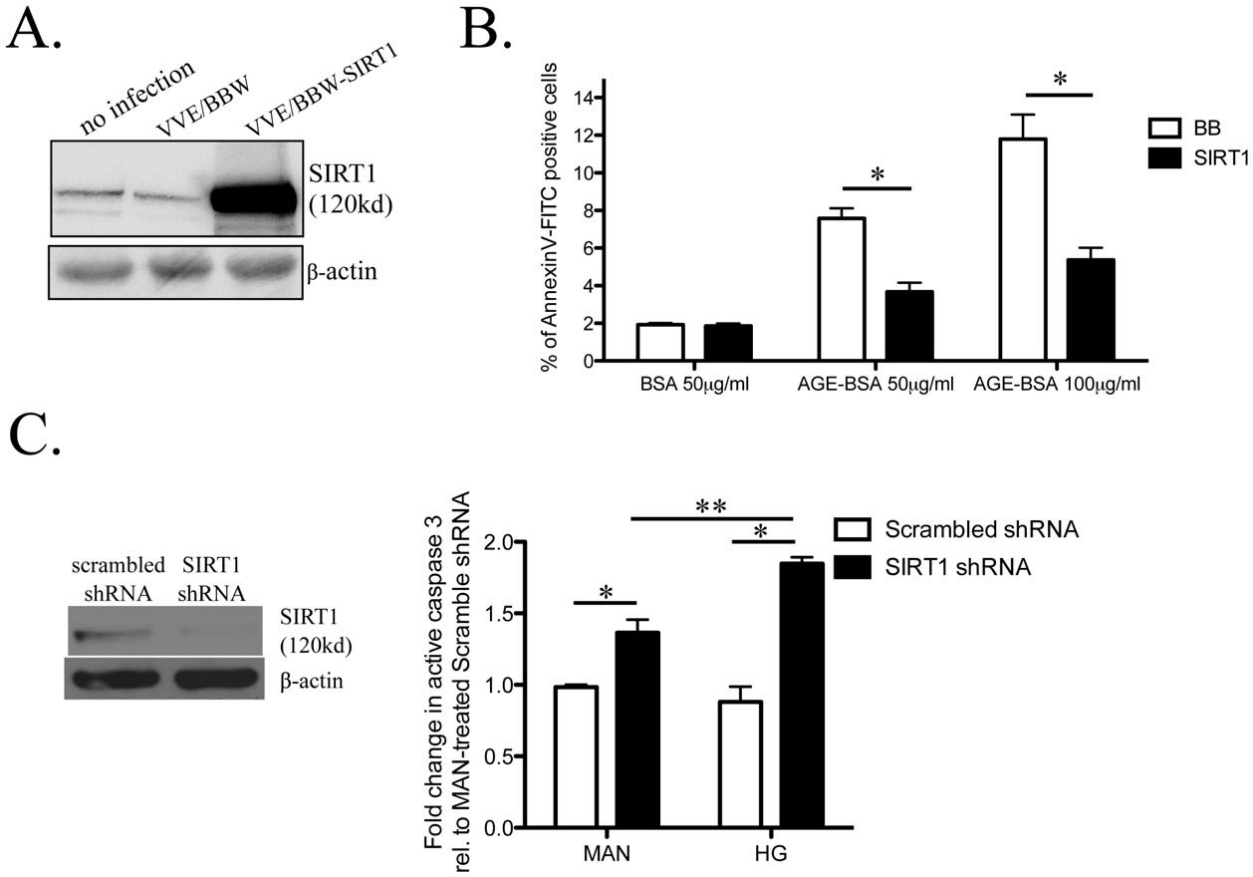
Sirt1 overexpression and knockdown on podocyte apoptosis. The expression of Sirt1 in murine podocyte without infection (no infection), infected with the empty lentivirus VVE/BBW (BB), or infected with the VVE/BBW-Sirt1 lentivirus with stable overexpression of Sirt1 (Sirt1) was confirmed by Western blotting (A). Apoptosis of BB (□) and SIRT (▪) podocytes treated with BSA (50 µg/ml) or AGE-BSA (50 and 100 µg/ml) for 16 h were quantified by flow cytometry after annexin-V-FITC labeling (B). *p<0.05, n = 3. Murine podocytes tranduced with either a scrambled shRNA or Sirt1-targeting shRNA were treated with either 30 mM of mannitol (MAN) or glucose (HG). Knockdown of Sirt1 expression was confirmed by Western blotting (C). Fold change in active caspase compared to scrambled shRNA podocytes treated with MAN is shown. *p<0.05, n = 3.

To determine the role of Sirt1 on AGE-BSA-induced podocyte apoptosis we over expressed Sirt1 by lentiviral transduction of podocytes. Stable expression of Sirt1 in blasticidin-selected cells was confirmed by Western blotting (Fig. 5A). Infection and selection of stably transduced podocytes were repeated several times and these cells were used in subsequent experiments. Sirt1 and BB podocytes were exposed to AGE-BSA at either 50 µg/ml or 100 µg/ml or to BSA at 50 µg/ml as control. Percentage of apoptotic cells was quantified by flow cytometry after annexin V FITC labeling. Podocytes with Sirt1 over-expression were protected from AGE-BSA-induced apoptosis (Fig. 5B). The percentage of annexin V-FITC positive cells was significantly higher in BB podocytes exposed to AGE-BSA than Sirt1 podocytes: 7.6±0.27% *vs.* 3.8±0.28% for AGE-BSA 50 µg/ml and 11.8±0.75% *vs.* 5.4±0.37% for AGE-BSA 100 µg/ml (Fig. 5B).

### Hyperglycemia on Sirt1-dependent podocyte apoptosis

To assess the role of Sirt1 on hyperglycemia-induced podocyte apoptosis, we transduced podocytes with either a scrambled or a Sirt1-targeting shRNA. Sirt1 knockdown was confirmed by western blotting. Apoptosis was assessed by ELISA measurement of active caspase 3 in podocytes transduced with either scrambled or Sirt1-targeting shRNA. Transduced podocytes were treated with either 30 mM mannitol (MAN) or D-glucose (HG). HG treatment without Sirt1 knockdown did not significantly increase active caspase 3 (Fig. 5C). HG treatment, as compared to MAN treatment, of podocyes with Sirt1 knocked down caused more significant increase in activated caspase 3 (1.85±0.05 vs. 1.36±0.09 **p<0.05 for both). Of note, Sirt1 knockdown in podocytes increased active caspase 3 regardless of treatment conditions (1.36±0.09 vs 0.98±0.02 in MAN and 1.85±0.05 vs 0.88±0.11 in HG. *p<0.05).

### Sirt1 expression is reduced, Bcl2l11 expression is increased, and Foxo4 acetylation is increased in diabetic *db/db* mice

**Figure 6:**
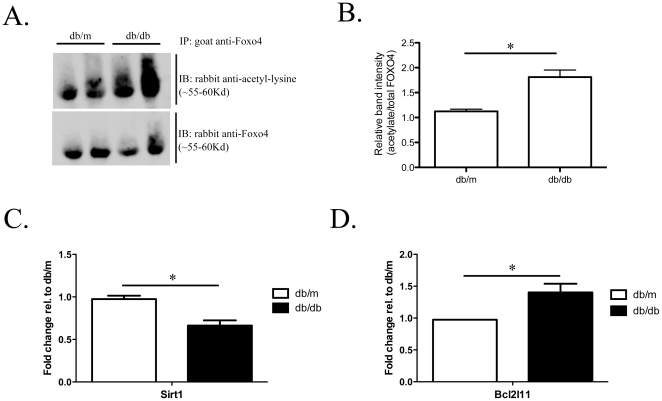
Acetylation of Foxo4 and expression of Sirt1 and Bcl2l11 in isolated glomeruli of *db/db* and *db/m* mice. A representative Western blot of glomerular protein lysates from *db/db* and *db/m* mice at 7–8weeks of age were immunoprecipitated with an anti-Foxo4 antibody and immunoblotted with an anti-acetyl-lysine antibody (A). Immunoblotting for total Foxo4 was used to normalize for sample loading and IP efficiency. The ratio of band density for acetyl-lysine Foxo4 and total Foxo4 (acetylated/total Foxo4) is presented as relative Foxo4 acetylation (B). Relative band intensity is significantly higher in *db/db* than *db/m* (1.811±0.143 *vs* 1.123±0.04, *n* = 4 per group, *p*<0.05). Glomeruli were isolated from diabetic *db/db* and non-diabetic *db/m* mice at 11–12 weeks of age. mRNA expression of *Sirt1* (C) and *Bcl2l11* (D) were quantified by real time PCR. Data is presented as fold change in gene expression relative to *db/m*. (*n* = 7 in *db/m* group; n = 5 in *db/db* group). *p<0.05.

Since AGE enhances Foxo4 acetylation in cultured podocytes and the diabetic milieu is characterized by increased AGE accumulation, we examined the acetylation of Foxo4 in isolated glomeruli from diabetic animals. Glomeruli from diabetic *db/db* mice and non-diabetic *db/m* mice on the C57BLKS background between 7 to 8 weeks of age were isolated by Fe_3_O_4_ perfusion. We had chosen to examine Foxo4 acetylation in the glomerular isolates from mice between 7 to 8 weeks of age because podocyte apoptosis in *db/db* mice has been previously reported to peak at approximately 8 weeks of age and then declines thereafter [7]. Foxo4 acetylation was detected by immunoblotting for acetylated lysine in anti-Foxo4 antibody immunoprecipitated glomerular protein lysates. Foxo4 acetylation is significantly higher in *db/db* compared to *db/m* mice (Fig. 6A and 6B). The expression of Sirt1 is reduced and of Bcl2l11 is increased in isolated glomeruli of *db/db* mice compared to their non-diabetic *db/m* littermates that are between 11 to 12 weeks of age (Fig. 6C and 6D).

## Discussion

Protein lysine acetylation of histones is a key posttranslational modification that affects chromatin structure and contributes to the transcriptional regulation of gene expression. Acetylation of other nuclear transcriptional regulators, such as general transcription factors and site-specific DNA binding factors, has also been shown to regulate protein-DNA and protein-protein interactions, thereby regulating gene transcription [25]. Although several lines of evidence indirectly support the link between histone and transcription factor acetylation in the pathogenesis of diabetes [26], the role of transcription factor acetylation on the pathogenesis of kidney disease in diabetes has never been established. Our results provide the first direct evidence that an alteration in the acetylation of a transcription factor (Foxo4) in the podocyte promotes the transcription of a pro-apoptotic gene, Bcl2l11 and contributes to podocyte loss, which is considered a key mechanism of diabetic nephropathy.

Acetylation of nuclear transcription regulators has an important role in diabetic kidney disease. In addition to Foxo, several key transcription factors that are known to play a role in podocyte apoptosis and activated in diabetic nephropathy (i.e. p53 [27], [28], TGFβ/SMAD [29], [30], and Jak/Stat [31]) are regulated by acetylation and targeted by Sirt1 for deacetylation. Even though our study suggests that Sirt1 expression and Foxo4 acetylation is altered in diabetes, we have not excluded the possibility that other targets of Sirt1 might also play a role in podocyte apoptosis. For instance, Sirt1 has been shown to de-acetylate p53 in cultured murine kidney mesangial cells and attenuate oxidative stress-induced apoptosis [32]. In fact, there is much similarity shared by p53 and Foxo's with regards to regulation by post-translational modification (acetylation and phosphorylation), effects on cell cycle regulation and apoptosis, and deacetylation by Sirt1 [33]. Furthermore, there is significant cross talk between Foxo and p53 [33] and other transcription factors [34]. Transcription factors that are targeted by Sirt1 and also play a significant role in the pathophysiology of kidney disease include Smad7 [35], HIF-2α [36], and Stat3 [37]. The role of Sirt1-mediated deacetylation of p53, Smad7, HIF-2α and Stat3 in kidney diseases deserves further investigation in the future.

Although much is known of histone acetyltransferases (HAT)-mediated change in the acetylation status of nuclear protein, the role of protein deacetylases is less well characterized. In this study, we found that the increase in Foxo4 acetylation is linked to a reduction in the expression of the Sirt1 protein deacetylase. Sirt1 is known to be involved in cellular resistance to metabolic, oxidative and hypoxic stress, DNA damage repair, gene transcription, apoptosis and beneficial effects of caloric restriction. Hao and Hasse recently reviewed the relevance of SIRT-dependent pathways on renal physiology and kidney diseases [38]. Sirt1 expression is increased in conditions where there is a reduction of energy/nutrient (i.e. starvation, caloric restriction) or acute oxidative stress. We suspect that accumulation of AGE in the diabetic milieu contributes to the suppression of Sirt1 expression. However, the exact mechanism of AGE-mediated downregulation of Sirt1 remains to be identified. The regulation of Sirt1 expression occurs at both the transcriptional and translational levels: de-repression of Sirt1 transcription by a hypermethylated-in-cancer1:C-terminal binding protein repressor complex occurs in response to oxidant stress [39], phosphorylation of a mRNA binding protein, HuR, reduces the stability of Sirt1 mRNA [40], and p53-induced expression of miR34a represses Sirt1 expression [41]. Since AGEs promote the generation of reactive oxygen species by activation of NADPH oxidase [42] and p53 activation is linked to oxidant stress [43], we suspect that AGE-mediated downregulation of Sirt1 likely occurs through an oxidant-dependent mechanism. Although it has been shown previously that an increase in Sirt1 expression and the interaction of Foxo and Sirt1 in response to oxidative stress is thought to “tip the balance towards cell survival” by shifting the expression of Foxo target genes from apoptosis response to oxidative stress resistance [44], this salutary effect of Sirt1 on cell survival appears to be lacking in the diabetic milieu where the podocytes are known to be apoptosis prone and oxidatively stressed.

Post-transcriptional modifications are important regulators of Foxo's transcriptional activity. We have focused on the acetylation of Foxo4 in the present study since it is known that Sirt1 de-acetylates Foxos and protects cells from oxidant stress and apoptosis [12]. We previously shown that AGE reduced the phosphorylation and increased the nuclear localization of Foxo4 in cultured podocytes [9].

We had previously speculated that AGE-mediated down regulation of Sirt1 would promote the acetylation of Foxo and negate Foxo-mediated the protection against oxidant stress and apoptosis. Although we observed that the expression of *Bcl2l11* is increased by AGE [9] and the expression of a Foxo-target gene involved in oxidant stress response—catalase—is suppressed by AGE, we have not been able to demonstrate a significant change in the Foxo4 binding to the catalase promoter in response to AGE treatment (unpublished data). We suspect that AGE-mediated suppression of catalase is likely mediated through other members of the Foxo family or other transcription factors.

We also found that hyperglycemia induces activation of caspase 3, however, only when Sirt1 expression is reduced. This suggests that hyperglycemia by itself is probably not enough to induce podocyte apoptosis. . However, when Sirt1 expression is reduced by shRNA, HG caused a significant increased in activated caspase 3 (Fig. 5c). These observations suggest that high glucose could increase podocyte apoptosis when Sirt1 is suppressed by excess of AGE. This scenario of hyperglycemia and SIRT1 suppression likely occurs in the diabetic milieu where prolong hyperglycemia leads to AGE formation, and AGE reduces *SIRT1* expression. The relatively short treatment duration with high glucose in our experiment could explain the lack of caspase 3 activation.

The importance of the Sirt1-Foxo4-Bcl2l11 pathway we described here is consistent with the findings of Kume *et al*, where they identified a role for Sirt1 in aging-related nephropathy affecting the renal cortex [45]. Although we were unable to detect a significant change in Sirt1 in the tubulointerstitial compartment of DN, others have reported that the expression of another member of the sirtuin family, Sirt3 is suppressed in cultured renal tubular epithelial cells by angiotensin II, a key mediator of the pathophysiology of diabetic renal disease [46].

Efforts to increase Sirt1 activity by resveratrol treatment [47] or to increase Sirt1 expression in transgenic mice by knockin [48] or over expression of a bacterial artificial chromosome containing a Sirt1 transgene [49] have been shown to improve glycemic response in murine models of diabetes mellitus. However, in order to further define the role of Sirt1 on podocyte apoptosis in DN and avoid potential confounding effects from improved glycemic control in these Sirt1 over expression models, studies that examine transgenic animals with podocyte-specific Sirt1 over expression and diabetes will need to be carried out.

We demonstrated in this study that alteration in the acetylation status of a transcription factor, Foxo4, is linked to a reduction in the expression of Sirt1 and contributes to the development of podocyte loss in diabetes. These findings highlight the importance of acetylation of nuclear factors in the regulation of gene expression in an important disease process. A recent study has shown that protein lysine acetylation is not only limited to nuclear transcription regulators, but also cellular enzymes that catalyze intermediate metabolism and plays a major role in metabolic regulation [50]. The reduction of Sirt1 and associated change in the status of lysine acetylation could have broad effects ranging from gene expression to enzymatic activity. Pharmacologic intervention to normalize Sirt1 expression or prevent acetylation of Foxo4 should be explored as potential approaches to improve the outcome of diabetic kidney disease.
